# Community readiness and momentum: identifying and including community-driven variables in a mixed-method rural palliative care service siting model

**DOI:** 10.1186/s12904-018-0313-5

**Published:** 2018-04-06

**Authors:** V. A. Crooks, M. Giesbrecht, H. Castleden, N. Schuurman, M. Skinner, A. Williams

**Affiliations:** 1 Department of Geography, Simon Fraser University, 8888 University Drive, Burnaby, BC V5A 1S6 Canada; 2 Department of Geography and Planning and Department of Public Health Sciences, Queens University, 62 Fifth Field Company Lane, Kingston, ON K7L 3N6 Canada; 3 Trent School of the Environment, Trent University, 1600 West Bank Drive, Peterborough, ON K9L 0G2 Canada; 4 School of Geography & Earth Sciences, McMaster University, 1280 Main Street West, Hamilton, ON L8S 4M1 Canada

**Keywords:** Palliative care, Canada, Rural, Community, Service siting

## Abstract

**Background:**

Health service administrators make decisions regarding how to best use limited resources to have the most significant impact. Service siting models are tools that can help in this capacity. Here we build on our own mixed-method service siting model focused on identifying rural Canadian communities most in need of and ready for palliative care service enhancement through incorporating new community-driven insights.

**Methods:**

We conducted 40 semi-structured interviews with formal and informal palliative care providers from four purposefully selected rural communities across Canada. Communities were selected by running our siting model, which incorporated GIS methods, and then identifying locations suitable as qualitative case studies. Participants were identified using multiple recruitment methods. Interviews were transcribed verbatim and the transcripts were reviewed to identify emerging themes and were coded accordingly. Thematic analysis then ensued.

**Results:**

We previously introduced the inclusion of a ‘community readiness’ arm in the siting model. This arm is based on five community-driven indicators of palliative care service enhancement readiness and need. The findings from the current analysis underscore the importance of this arm of the model. However, the data also revealed the need to subjectively assess the presence or absence of community awareness and momentum indicators. The interviews point to factors such as educational tools, volunteers, and local acknowledgement of palliative care priorities as reflecting the presence of community awareness and factors such as new employment and volunteer positions, new care spaces, and new projects and programs as reflecting momentum. The diversity of factors found to illustrate these indicators between our pilot study and current national study demonstrate the need for those using our service siting model to look for contextually-relevant signs of their presence.

**Conclusion:**

Although the science behind siting model development is established, few researchers have developed such models in an open way (e.g., documenting every stage of model development, engaging with community members). This mixed-method study has addressed this notable knowledge gap. While we have focused on rural palliative care in Canada, the process by which we have developed and refined our siting model is transferrable and can be applied to address other siting problems.

## Background

Reflecting a demographic trend witnessed across much of the Global North, Canada is experiencing rapid population aging. According to our last national census, an estimated five million Canadians were 65 years of age or older and this number is expected to double in the next 25 years, reaching over ten million by 2036 [1]. By 2051, about one in four Canadians will be over the age of 65 [1]. This rate of aging, however, is not uniform across the country. Geographically, it is Canada’s rural communities that are experiencing the most rapid rates of aging when compared to their urban counterparts [2]. This is largely due to local rural residents ‘aging in place’, but also to senior in-migration that is occurring in 16% of Canadian rural communities due to factors such as affordability and lifestyle [2, 3]. This rapidly aging rural population is a timely concern with regard to the provision and receipt of palliative care as those aged 65 years and over account for over 75% of the total deaths in Canada each year [4]. Thus, a fast-approaching reality is that health service providers in rural Canadian communities are facing heightened demand for palliative care provision while simultaneously coping with shrinking budgets and in many cases health care restructuring that is affecting service provision [5, 6].

Recognition of the impending growing demand for palliative care has gained attention by provincial, territorial, and municipal governments across Canada [7, 8]. For example, it has been recognized that Canada is facing a scarcity of such services with the gap between demand and service availability continuously expanding [9–12]. Despite this attention, little consideration has been given to the differential geographic, resource, and population contexts that exist between rural and urban service provision and availability [13]. It is within the rural palliative care context that health care decision-makers are faced with the challenge of meeting both immediate and long-term palliative care needs for a relatively small and sparse population dispersed across a vast landscape while at the same time rationing palliative care service allocation due to limited funding and budget allocations [14]. As a result, more rational, systematic and evidence-informed methods to determine how best to deliver the most effective and efficient care to the greatest number of people are significantly needed. The ability to base such decisions on results generated from evidence-informed, rational, and systematic methods enhances the credibility of such insights and facilitates a more transparent decision-making process. Acknowledging this, policy-makers and administrators are actively seeking such approaches to decision-making that integrate best practices and evidence of effectiveness [15, 16].

**Fig. 1:**
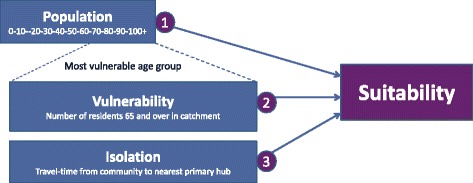
Initial Siting Model Proposed Before Pilot Study Interviews (adapted from [38])

Here we offer a novel approach to supporting health service policy-maker and administrator decision-making regarding enhancing palliative care in rural Canadian communities through the development of a mixed-method service siting model tailored to this context. In doing so, we acknowledge that this service-siting approach serves as only one component of a suite of activities or actions that can lead to more equitable palliative care access and provision in rural Canadian communities. We previously ran a pilot study in the Canadian province of British Columbia (BC) that resulted in the creation of a mixed-method siting model [17]. This siting model is summarized in Figure 1. Building from our earlier pilot study, where we developed a preliminary siting model using data focused specifically on BC, here we report on the findings of qualitative interviews conducted with key informants (*n* = 40) in four purposefully selected rural communities across other parts of Canada to assess the appropriateness of the siting model for other rural palliative care service contexts. An important finding of these interviews is that the way in which some of the variables associated with the ‘community readiness’ component in the existing model, a variable we introduce in greater detail below, need to be altered to be appropriate for a wider range of rural Canadian communities. In this article we examine this finding, justifying a necessary alteration to the siting model using first-hand insights offered by the key informant interview participants. Ultimately, we introduce an important adjustment to the siting model as it was previously reported. In the discussion section we contend that, based on these findings, some aspects of assessing ‘community readiness’ for enhancing palliative care in rural areas must be assessed qualitatively or subjectively and in ways that allow for local context to be considered and propose ways for doing this. First, however, in the following sections we provide a brief review on the use of siting models in health care decision making before providing a summary of our pilot study and key findings.

### A siting model approach to health care decision-making

Although geographic access does not necessarily equate full health care accessibility, it is a critical prerequisite to enabling equitable care provision [18], including palliative care. As such, a primary concern for decision-makers is determining how to make health care services geographically accessible (e.g., available locally or within a reasonable transit time). Achieving geographic accessibility is particularly problematic in Canada, where rural regions account for approximately 90% of the total land mass and about one third of the total population [19]. Considering this geography, and the existing financial/resource limitations, it is simply not feasible to enhance services in all rural and remote communities [20]. Thus, what is needed is the thoughtful identification of communities that are most suitable for targeting enhancement strategies. A spatial decision support system, or siting model, can assist with this task and can serve as a valuable tool for regional health service planning.

Based on Geographic Information System (GIS) technology, siting models are tools designed to collect, integrate, and model spatial data [21]. Use of such a tool can allow healthcare decision-makers to explore spatial questions, like: where would it be best to site or enhance a specific health service to most effectively serve a dispersed population? The development of siting models provide decision makers with the ability to access relevant data from existing datasets, and sometimes even collect new primary data (as is the case with the current siting model), and model prospective service configurations within and across their regions [14, 22–24]. Thus, siting models hold the potential to provide systematic support to decision-makers as they tackle challenging service siting questions.

While there exists a significant amount of research on the science of siting and model development [23, 25, 26], there are few accounts on the development of specific health service siting models within academic literature [27–30]. The paucity of such published accounts points to the highly privatized use of such models, whereby it is generally those who can afford to pay for such skills and services who are creating and using siting models (e.g., retailers, private hospitals, other corporations). There are, however, some articles and reports that document the development of siting models for use in health services. One such example involves the development of an MRI site in central Newfoundland & Labrador, which is a province in Atlantic Canada [31]. Having no expertise in siting analyses or access to siting model supports, a research team sought to create their own siting model to determine which out of two hospitals would be the most viable host for an MRI. Four variables were considered in their model: access, demand, cost/benefit, and human resources. The findings from their model facilitated the final decision and successful approval for the MRI site development [32]. A second example pertains to the development of a siting model to assist with determining where to site a new acute care hospital in Durham, which is in Northern England [33]. A regional health authority in Durham commissioned a report to characterize the optimal spatial pattern of future hospital development, which was accomplished through considering the adequacy of existing facilities, spatial distribution of potential users, and optimal locations of future services. Relying heavily on spatial statistics, the results of this model recommended a site located within close proximity to another regional health authority.

Despite siting models having been recognized as effective tools for health service decision-makers in various fields, including palliative care, there is little literature for those outside the private realm, who may not be able to afford to commission such projects, to draw from that captures the *rationale* for the inclusion of particular variables in existing models. This limits the transferability of existing models, including the two summarized above, to other contexts. Our research team set out to address this gap by developing a mixed-method siting model that is made freely available, and which offers a sound rationale for the inclusion of each variable and in doing so draws on qualitative insights and existing population-level datasets. As a case study, the focus of our model is on rural palliative care service delivery in Canada. This model can be used by health service administrators to determine which rural communities are most in need of enhancing, and also most ready to enhance, their local palliative care service provision. As we have argued elsewhere, having access to such a tool is an invaluable asset for administrators, decision-makers, and other local community members who are interested in enhancing and expanding upon existing rural palliative care services in Canada [34]. We contend that by sharing details of the development of this model and the rationale for each variable included within, all or part of the model can be assessed for transferability for use in other geographic or health service contexts.

### Our pilot study

The analysis presented herein stems from a previously conducted pilot study that aimed to identify communities in rural and remote areas of the province of BC that were most ready to enhance their existing palliative care services [17, 35]. Communities were identified through a siting model that was created to consider palliative care-specific locational factors. Running the model results in identifying communities both ready for and in need of service enhancement in a defined area, but it does not determine the nature of enhancements that are needed nor does it consider cost-based factors as these are outside the scope of the model. Thus, our pilot study created a locational service siting model specific to rural palliative care in BC that ranks communities in terms of their suitability for service enhancement.

Our pilot study began by conducting a spatial analysis to determine the geographic accessibility of existing specialized palliative care services in BC. We did this in order to exclude communities with specialized palliative care on-site. A location analysis model was then developed using GIS, building upon the work of Schuurman et al. [36, 37], to assess the suitability of communities not excluded in this first step to rank their suitability for enhancing palliative care services. This location analysis model involved the following factors: (1) the total population within one-hour of the communities (whereby larger populations were deemed more suitable); (2) the vulnerability of the community, which was calculated as the total population 65 years of age and older within the one-hour area (whereby the larger older populations are more suitable); and (3) the isolation of the community as measured by travel time to the nearest specialized palliative care facility (whereby longer travel times to existing facilities are more suitable). Nineteen communities across rural and remote BC were identified using the GIS location analysis model, providing valuable information regarding which communities are most in need of palliative care service enhancement by ranking them based on the score generated from the four weighted factors [38].

**Fig. 2:**
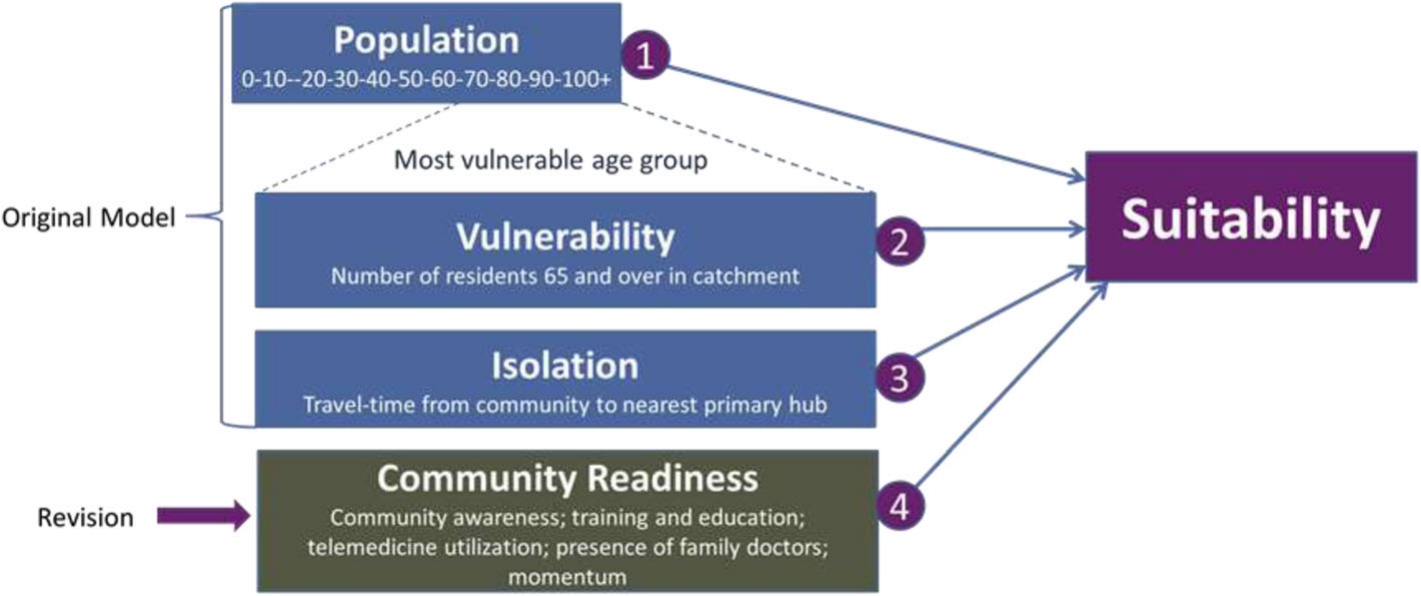
Revised Siting Model Based on Pilot Study (adapted from [41])

After running the service siting model, the next phase of our pilot research involved conducting interviews with key informants in a cluster of three communities ranked highly by the model to gather information to assist with refining the model and to assess whether or not local experts agreed that they were ready for and in need of palliative care service enhancement. We conducted 31 semi-structured interviews with key informants in the BC communities of Trail, Nelson, and Castlegar. We pursued a number of qualitative analyses that assisted with better understanding the scope of palliative care need in rural BC and the implications of this for our siting model [see 34, 35, 39, 40]. Of relevance to the current article, it became apparent from the interview findings that an important additional factor needed to be added into the siting model: community readiness. As such, specific variables that collectively informed the communities’ readiness to enhance palliative care services were identified for inclusion [41]: (1) community awareness, (2) training and education, (3) telemedicine utilization, (4) presence of family doctors, and (5) community momentum. Figure 2 illustrates the revision to the original model and Table 1 provides details on these new variables.

**Table 1:** Community Readiness Variables and Indicators Added Following Pilot Study (adapted from [41])

Variable	Meaning	Binary Indicator (Y/N)
Community Awareness	Showing evidence that palliative care is a priority issue. Does palliative care have visibility or a ‘profile’ in the community?	Is there a local hospice society (in that these groups play a major role in local advocacy)?
Training and Education	Strengthening palliative care in rural communities requires providing local education opportunities. Is there a site to host and possibly coordinate such initiatives?	Is there a local college or university campus?
Telemedicine utilization	Telemedicine can increase capacity for providing palliative care in smaller sites. Is the community ready to link to larger centres via telemedicine in order to facilitate information sharing?	Is there regular use of telemedicine at the local hospital?
Presence of family doctors	Family doctors play a vital role in providing palliative care in rural areas. Are there adequate family medicine resources locally to enhance palliative care provision?	Do family doctors practicing locally have an adequate family physician to population ratio?
Momentum	Enhancing palliative care is not an end point, but rather the start of accomplishing larger goals. Has there been demonstration by the community of the desire to increase palliative care capacity?	Has a proposal been put forth to create a local hospice?

In order to be integrated into the siting model, indicators of each of the identified community readiness variables were developed based on the results of the key informant interviews to be measured as binary yes or no answers for inclusion in the siting model. For example, the *community awareness* variable was considered to be a yes if a hospice society was in operation in the community, which was determined by looking for hospice or palliative care societies in each of the communities using online search engines. The *community momentum* variable was considered to be a yes if a proposal had been developed with the intention of creating a hospice residence in the community, which was determined by contacting and asking local hospice societies. Yes answers received a .20 score and no answers received a 0 score, which ensured that the maximum score possible for the community readiness arm of the model was 1, which is the same total maximum score for each of the other arms. Readiness scores were tabulated by adding the results of each of the readiness indicators together and adding this score with the previous suitability score in the mixed methods location analysis model. After including the community readiness arm in the model we ran the revised siting model in BC to see if this change made any difference in the suitability rankings assigned to communities [41]. Although the differences were minimal, we believed inclusion of the community readiness variables was of great importance as they offer a ‘grounded’ perspective, which produces a more refined and robust assessment of a community’s suitability and readiness to enhance their palliative care service provision. In the present article we focus once again on these community readiness variables, refining them based on the findings of a new study that has involved applying the siting model across Canada and conducting new key informant interviews in four more rural communities based in other provinces.

## Methods

Since completion of the pilot, the research team has now embarked on a national study, which employs the mixed-methods location analysis model across Canada, excluding the province of BC (where it was originally tested), to conduct further model testing and refinement. Based upon the pan-Canadian results, the model showed that 58 communities were identified as highly suitable for enhancing their palliative care services. We next pursued identifying four case study communities from these 58 for further qualitative research. To do this, we narrowed our focus to communities identified in the provinces of Alberta, Manitoba, Ontario, and Newfoundland & Labrador. These provinces were selected based on their geographic and population diversity. For the four provinces, contextual information about each identified community was garnered (e.g., percent of population employed in agricultural, percent of population self-identified as Aboriginal, etc.) to inform the selection of the qualitative case-study communities that would represent socio-economic and demographic diversity found across Canada while not replicating the profile of those included in the initial BC-based pilot study. It was from this contextual information that the four case-study sites reported on here were purposely selected by our research team: (1) Lloydminster, Alberta/Saskatchewan (an agricultural town with a population of just over 30,000); (2) Thompson, Manitoba (a resource town with a population close to 13,000); (3) Fort Frances, Ontario (a fishing and milling town with almost 8000 residents), and; (4) Happy Valley-Goose Bay, Newfoundland & Labrador (a small town home to a military base with just over 7500 residents).

Following ethics approval from Simon Fraser University and the regional ethics boards for the health authorities that oversee these communities, semi-structured phone interviews were conducted with formal (*n* = 34) and informal (*n* = 6) palliative care providers and administrators across of the four case study communities listed above (10 interviews per community for a total of 40 interviews). Participants were identified through online directories of health service employees, hospice contact information, advertisements in the local paper, and study information that was circulated by some health authorities. Lasting on average 74 min, the interviews inquired into participants’ experiences with: palliative care provision; important community characteristics; community health and health care priorities and challenges; community need for palliative care and existing availability; and their perspective on our siting model approach.

All interviews were digitally recorded, transcribed verbatim, and entered into NVivo™ software for data management. Selected transcripts were reviewed by members of the investigative team to identify emerging themes and determine the scope and scale of each. Following this, a coding scheme was developed to capture these themes as well as organize contextual information shared by participants. Thematic analysis ensued on the data set, using investigator triangulation to confirm the assignment of codes to data segments, where emergent patterns in the data were categorized [42]. The emergent patterns were then reviewed, refined, and compared and contrasted to the pilot study findings. It was through this process that the refinement to the community readiness component of the model was identified as a necessary outcome of the interview findings, which is what we report on in the section that follows.

## Results

While the pilot study findings pointed to the need to include community readiness as a variable in the siting model, analysis of the data from this new, pan-Canadian, dataset revealed inconsistencies in how to conceptualize this variable. Participants’ comments reveal that an inherent challenge exists in accurately pre-determining binary indicators that capture contextually-drive qualitative aspects of the model like community readiness, such as community awareness and momentum. As such, our national study findings indicate that further refinement of the model is necessary to more effectively capture these complex and context-driven factors, breaking them out of the binary ways they were initially summarized in Table 1.

In this section we characterize the breadth of factors that illustrate the presence of community awareness and momentum around building capacity for palliative care in rural Canadian communities from across the four case study communities. Specifically, the interviews point to factors such as educational tools, volunteers, and local acknowledgement of palliative care priorities as reflecting community awareness and factors such as new employment and volunteer positions, new care spaces, and new projects and programs as reflecting momentum. We expand on these factors here in order to assess their scope and demonstrate their relevance to enhancing palliative care in rural communities. In the discussion section that follows we point to the fact that the breadth of factors identified in the current study and our earlier research collectively show that community awareness and momentum variables in the siting model must be assessed subjectively and we consider how this can be done.

### Community awareness: Does palliative care have visibility in the community?

During the interviews, it became apparent that participants were aware of local palliative care provision and felt it had a visible presence in each of their four communities. While this may have been confirmed via the original siting model indicator of whether or not there was a local hospice society, other indicators emerged that were equally capable of demonstrating local awareness regarding the importance of palliative care in the community. Specifically, the new indicators that emerged include the presence of: (1) educational tools that promote palliative care awareness and knowledge; (2) community-based palliative care volunteers; and (3) local stakeholder acknowledgement that palliative care is indeed prioritized.

#### Educational tools that promote local palliative care awareness and knowledge

Review of the transcripts revealed that some community members were actively involved in the development of local palliative care educational tools and training opportunities that aimed to increase awareness about, and visibility of, palliative care. For example, in Happy Valley-Goose Bay, a health care provider described how she, along with two other health workers, developed a palliative care information booklet to disseminate to clients and families. This participant explained how the purpose of the booklet was to make information about palliative care accessible for the local populations by avoiding medical jargon and keeping the information “*as simple and as precise, but true to life, for the palliative care patient and their family so that they’d have an understanding of what was going on.”* Some participants described the existence of palliative care training opportunities, such as the development of “*a training session for the nursing staff working on our palliative care beds, so they’ve actually had some education just recently”* in Lloydminster. Other participants spoke to how they had developed regional interdisciplinary committees to identify gaps in palliative care and develop educational sessions. Furthermore, because many Aboriginal elders die in the hospital in Fort Frances, it was explained that health care administrators have:

…*made it a priority to train and educate [hospital] staff on cultural competencies and all of that because they recognize that that is needed, so they are working with some First Nations [Aboriginal] organizations to try to develop … more training and materials around that for their staff.*

Such findings demonstrate how community awareness of palliative care operates, and is being further promoted, through the existence of locally developed educational tools and training sessions.

#### Community-based palliative care volunteers

Across the case study communities it was found that volunteers formally and informally facilitated palliative care provision in their communities, offering various means of support for community members and their families facing a life-limiting illness. Some participants explained that due to their ‘close-knit’ community culture, in some cases a whole town would informally pull together to ensure families received the care they needed at the end of life. However, in other communities, more formal volunteer groups existed. A participant from Lloydminster described the important role that volunteers play:

*My volunteers help with driving people to appointments, doing some running around, friendly visiting…at the hospital we have three palliative care rooms and they’re checking, make sure there’s coffee and that kind of thing as well as visiting patients and families.*

It was often explained that volunteers were mainly responsible for assisting patients with transportation, meals, and emotional/psychosocial support by visiting with patients and their families. The existence of such formal and informal volunteers in the communities demonstrates community awareness about palliative care and the identified need to enhance formal existing services.

#### Stakeholder acknowledgement that palliative care is a local priority

Although not always in the spotlight, many participants (as local stakeholders in palliative care) described feeling that members of their communities were aware of palliative care and even the need for service enhancement. As this participant from Fort Frances described: “*I think it’s [palliative care] one of their big priorities, they are as a community very very good at providing excellent palliative care”, while another explained that* “*I think there is an increasing awareness of the need for providing palliative care and what that means.”* Regarding existing conversations on and awareness about the need to enhance local palliative care, this Fort Frances participant stated that:

*there is the awareness and there are people coming together to try to provide appropriate palliative care…so the desire and the will is there and people realize that it’s something that we have to continue to look at and develop and grow, to meet the needs of the community.*

These descriptions highlight local awareness about palliative care and the importance it carries for their communities. Participants also commented on how regional and provincial changes to palliative care policies and programs sometimes created new local awareness of palliative care through conversation among stakeholders. In the case of Thompson, a new advanced care planning strategy led to the development of a new program involving conversations between “*a social worker, or somebody trained, going in and talking to somebody whose still mentally alert…transcribe their conversation about who they are and their life and the things they would like to pass on to their family.”* Taken together, such awareness of the local prioritization of palliative care among stakeholders illustrates one way in which such care is viewed as a priority as well as wider community awareness.

### Momentum: Has there been a demonstration by the community of the desire to increase palliative care capacity?

Thematic analysis revealed that community momentum could not be captured through the sole binary indicator of whether or not a proposal for a hospice has been put forward in the community alone. While this factor does capture community momentum, it was found that other factors could similarly depict such desires to increase local palliative care capacity. These additional indicators include the local addition of new: (1) employment or volunteer positions; (2) rooms or spaces dedicated for palliative care; and (3) projects, plans, and programs.

#### Employment or volunteer positions

Some participants described how recently their communities had seen the addition of new palliative care employment or volunteer positions, which implies that the desire exists to enhance local palliative care services. Particularly in Fort Frances, where a participant described how they recently acquired “*a new nurse practitioner role for palliative care for the whole district…to support other Homecare providers in doing better palliative care.”* It was also mentioned that Fort Frances recently acquired a new palliative care coordinator for their district, which was described by a participant as someone who “*looks quite promising.”* While these new positions are the outcomes of regional priorities and their directives to enhance local palliative care services, other forms of momentum were found to be coming from the communities themselves. For example, in Fort Frances, there had previously been a strong palliative care volunteer group that had “*fizzled out*”; however, at the time of the interviews, a new volunteer coordinator was just assigned and was in the process of seeking out and training a new group of volunteers. She described:

*Our volunteers have to go through thirty hours of training before I can actually put them to work… It’s like a workshop thing… And then I’m going to be trying to get teaching sessions every couple of months or so somebody will come in and talk about this or that, Alzheimer’s or cancer or you know different aspects… But first I need the volunteers to be able to (chuckle) do the workshops.*

Although Fort Frances had not put forth a proposal for a new hospice to be created, the findings here depict a community with momentum with members who are actively trying to enhance their palliative care capacity through new employment positions and volunteers.

#### Rooms or spaces for palliative care

While the creation of a dedicated hospice for palliative care was beyond some rural communities’ capabilities, other types of momentum were occurring, particularly in the form of creating new rooms or spaces for palliative care. These rooms and spaces were often found in local hospitals, for example in Lloydminster, where a few participants described how their local hospital recently underwent renovations of three rooms to be set up solely for palliative care patients and their families. This participant stated that “*we’ve recently added on a palliative care room and renovated the other, like we used to have two palliative care rooms, we now have three and they’re linked together.”* It was found, however, that for some communities, it was simply not feasible to have dedicated palliative care spaces. Rather, they desired to have more flexible spaces that could accommodate those facing life-limiting illness and their families once the need emerged. This Fort Frances participant explained how they:

*are in the process of arranging for more or less carts so that we can turn any of the three acute care rooms and our two other sites into palliative care rooms. It’s a challenge when you only have three acute care rooms and our remote sites to designate one as a palliative care room. So we tend to try and do things a little bit differently and then theoretically any room in our long-term care facility should be able to accommodate palliative care*.

Taken together, these findings demonstrate that momentum is occurring in these communities through the creation of more palliative care rooms. However, each community addresses these needs in unique ways, thus reflecting how this indicator of momentum can only be interpreted within the context in which it is occurring.

#### Projects, plans, and programs

It became apparent that community momentum was being influenced by processes occurring at broader scales, like at the provincial and regional levels. This was found particularly in relation to Fort Frances, where participants described a new strategy that was in place by the Province of Ontario to enhance palliative care capacity. One participant explained the project, called *Advancing Integrative Palliative Care Services Across Ontario,* and how it aims to develop a plan for *“our Local Health Integration Network [LHIN] to say where there are gaps, this is our plan to fill them.”* More specifically, another participant explained that:

*the province has asked all of the local health integration networks to come up with palliative care and end of life plans for each one of the fourteen LHINs in the province. There are some LHINs that are further ahead than others…I believe that within our part of northwestern Ontario there is a strong desire to have a strong palliative care infrastructure in place as evidenced by at least our commitment to maintain that palliative care room in the hospital and have some resources at all of our sites to address palliative care service*.

Another participant from Fort Frances described how they were in the process of developing working groups to work on various projects, like “*coming up with common definitions and tools for palliative care, looking at caregiver supports, looking at provider education.”* At the time of the interviews, this project had just begun and demonstrated a change on the ground through the development of new jobs and volunteer positions in the community, which we discussed previously. As such, Fort Frances is an example of a community with momentum, moving towards enhancing their local palliative care services.

## Discussion

In this article we have presented a thematic analysis of 40 interviews conducted with formal and informal palliative care providers from four purposefully selected rural Canadian communities. The goal of these interviews was to gather first-hand insights from people involved in such care provision to identify if and how our existing service-siting model needs to be changed in order to enhance its utility across the diverse community types that make up rural Canada. We developed this model in an earlier pilot study as a way of identifying rural Canadian communities most in need of and most suitable for enhancing their palliative care service provision and conducted initial qualitative interviews about our model in the province of BC. From those pilot interviews we added a ‘community readiness’ arm to it, as shown in Figure 2 above. Table 1, above, summarizes the variables initially included in this arm of the siting model. The thematic analysis presented here shows that while community readiness remains an important factor in determining the suitability of a rural community for enhancing local palliative care provision, and thus should remain in the model, the way in which two of its associated variables—community awareness and momentum—are assessed must be revised.

Table 2 captures the revisions to the community readiness arm of the siting model informed by the thematic analysis presented herein. The model itself, shown in Figure 2 above, remains the same and the interviews confirmed the importance of including all previously-identified indicators of community readiness in the siting model. However, the way in which the presence or absence of momentum and community awareness are determined based on indicators has been revised using the input provided by the 40 interviewees. The findings show that these two variables cannot be captured using a single binary question as previously proposed by Crooks et al. [41]. Instead, someone applying the model will need to subjectively assess whether or not a community shows locally-relevant evidence of momentum and community awareness. In Table 2 we have provided examples of indicators for both of these variables based on the factors identified in the pilot interviews conducted in BC and the 40 interviews conducted in the current study. These examples, however, must not be thought of as exhaustive in that someone running the model must look for any and all evidence of what they can subjectively interpret as momentum or community awareness regarding palliative care in rural Canadian communities. We encourage those using or running the model to document this process and the sources consulted to justify how they have interpreted the presence or absence of these indicators. Here we have provided an example of consulting with community members to identify specific indicators. This could certainly be done elsewhere. Other potentially ripe sources of information could include community newspapers and newsletters, provincial health service policy documents, and attending hospital board or administrative meetings.

**Table 2:** Revised Community Readiness Variables and Indicators

Variable	Meaning	Indicator
Community Awareness	Showing evidence that palliative care is a priority issue. Does palliative care have visibility or a ‘profile’ in the community?	Subjective Indicator: presence of locally-relevant factors that indicate community awareness can result in ‘Yes’ for this variable (factors may include: presence of local hospice society; educational tools promoting awareness; presence of community volunteers; stakeholder acknowledgement).
Training and Education	Strengthening palliative care in rural communities requires providing local education opportunities. Is there a site to host and possibly coordinate such initiatives?	Binary Indicator (Y/N): Is there a local college or university campus?
Telemedicine utilization	Telemedicine can increase capacity for providing palliative care in smaller sites. Is the community ready to link to larger centres via telemedicine in order to facilitate information sharing?	Binary Indicator (Y/N): Is there regular use of telemedicine at the local hospital?
Presence of family doctors	Family doctors play a vital role in providing palliative care in rural areas. Are there adequate family medicine resources locally to enhance palliative care provision?	Binary Indicator (Y/N): Do family doctors practicing locally have an adequate family physician to population ratio?
Momentum	Enhancing palliative care is not an end point, but rather the start of accomplishing larger goals. Has there been demonstration by the community of the desire to increase palliative care capacity?	Subjective Indicator: presence of locally-relevant factors that indicate momentum can result in ‘Yes’ for this variable (factors may include: proposal for local hospice; new employment or volunteer positions; new spaces or places being created; projects and plans implemented at larger scales).

We noted previously that each of the four arms of the rural palliative care service siting model is assigned a maximum score of 1, and thus population, vulnerability, isolation, and community readiness are weighted equally in the overall suitability score provided. The rationale for this along with discussion regarding how to generate scores for the population, vulnerability, and isolation arms have been introduced elsewhere [14, 38] and are not re-examined here as our sole interest is in the community readiness arm. Following our revision to the indicators used to score each of the five components of community readiness based on the current analysis, as summarized in Table 2 below, three can be scored using binary Y/N questions while the remaining two require subjective assessment. Both the binary and subjective indicators will require obtaining information found online and/or from administrative databases (e.g., family physician practice information) and from local leaders in the palliative care community and so we contend that no new work is introduced by the revision to the siting model proposed here. After this information is gathered and reviewed, the subjectively assessed variables will have a value of .20 entered to indicate that, yes, that indicator is present in the community. Oppositely, a value of 0 will be entered if there is no subjective evidence of its presence. The binary indicators are objectively assessed and .20 is entered to indicate presence while 0 is entered to indicate absence.

### Strengths & Limitations

As the Canadian population ages at a rapid rate, growth in future demand for palliative care services cannot be ignored [43]. Here we have not taken a prescriptive approach to articulating how such services can be enhanced to facilitate greater and more responsive access to care. Instead, here we have focused on *where* to deepen service provision by developing a mixed-method service siting model that can assist with identifying which rural Canadian communities are most in need of and most suitable for enhancing their palliative care services. There are increasing calls for greater accountability with regard to health care spending, including in Canada [15, 16], and service siting models are a tool that can support this through making transparent how and why certain decision are made [44, 45]. Through documenting every step of the development and testing of the current model [14, 35, 38, 41], including in the current analysis, we believe that we have facilitated a high level of transparency that can be extended into its use in applied settings to enhance accountability. We also believe that the mixed-methods approach to this siting model that has incorporated qualitative and qualitative data throughout its development, wherein GIS-based location siting models typically rely on administrative data alone, also increases the transparency and accountability for two reasons. First, we have consulted with key stakeholders in several rural communities across Canada to garner their first-hand insights about factors they believe are important regarding the focus of our model to inform its refinement. Second, local consultation is required in using the model to assess the presence or absence of the community readiness variables, and especially those that have subjective indicators, which thereby necessitates local involvement in developing solutions for local needs.

Models of all forms are never perfect as they are based heavily on assumptions. The current siting model is no different. There are also limitations that are worth noting. For example, we equally weight each variable in the community readiness arm of the model as well as each of the four arms included in the model. While we did conduct earlier sensitivity analysis that showed that different weightings of the arm did little to change the overall suitability scores or ranking of communities against one another [41], this has not been done on the community readiness arm of the model. While the current data do not suggest any one community readiness variable to be more important than another, this is an aspect of our design that must be considered by those using the model in real-world settings. The siting model ultimately requires quantitative data to be entered and thus qualitative insights need to be quantified so that communities can be scored. This serves as a limitation in that factors that cannot be quantified cannot be incorporated. With regard to the current interviews, for example, we gleaned important insights about how boundaries and borders can create barriers or facilitators to palliative care service provision and access in rural Canada [46]. We could not link these insights to variables that can be quantitatively assessed and incorporated, and they are thus absent from the model as it is currently constructed. We also acknowledge that our model is focused heavily on formal palliative care provision as it is a service siting model and thus does not consider the full scope of supports that may be needed to facilitate rural-based end of life care more broadly. Finally, on a practical note, we conducted our interviews by phone and thus from a distance and so may have missed conversational nuances in facial expression or tone that would have been caught in person [47]. We were, however, quick to ask for elaboration or clarification in order to overcome this potential limitation as the cost-saving nature of this strategy is what enabled our inclusion of four very distant communities.

## Conclusion

Although the science behind siting model development has been documented in academic literature [23, 25, 26], few researchers have developed such models in an open way to address problems of health service siting [27–30]. This mixed-method study has addressed this notable knowledge gap through the detailed documentation of our siting model development, which aims to identify rural Canadian communities most in need of and ready to enhance their palliative care service provision. As we argued in the introduction, this is a needed and timely health service focus given the country’s aging population.

Our findings from 40 interviews with formal and informal palliative care providers across four rural Canadian communities demonstrate not only the value in, but also the importance of, incorporating community-driven indicators into service siting models as well as community consultation in the model development process. The findings also show that although the siting model we have developed requires the input of quantitative binary data, community readiness variables, such as momentum and awareness, must first be qualitatively assessed to garner more localized and context-specific information. Efforts to collect such data, for example via consulting with key-stakeholders, will ultimately contribute more ‘grounded’ knowledge to assist with identifying local needs, and ultimately, developing meaningful local solutions. Thus, our study emphasizes the importance of considering, including, and translating qualitative community-driven variables into quantitative indicators in order to produce more nuanced results that reflect the lived contexts of diverse communities.

While this study has focused on rural palliative care in Canada, the process by which we have developed and refined our siting model is highly transferrable and can be applied to address various other issues, in health services and beyond, within a diverse range of geographic contexts. For instance, our siting model could be adapted internationally for applications in jurisdictions where best practices in rural palliative care decision-making are in demand, such as in the Australian context [48]. GIS-based location service siting models can and do contribute valuable information to decision-makers by demonstrating how the place-based nature of health services can benefit from the visual capabilities of GIS [49]. For example, in our study, the resultant maps and spatial models are tools that can be used to translate numeric rankings into a visual format. This visualization of data holds the potential to assist those in policy to make informed, place-based decisions, while at the same time, facilitate the promotion of rational and transparent decision-making regarding the provision of services, within health care and beyond.

## Abbreviations

BC: British Columbia
GIS: Geographic Information Systems
